# Lifestyle, Eating Habits, and Health Behaviors Among Dietary Supplement Users in Three European Countries

**DOI:** 10.3389/fpubh.2022.892233

**Published:** 2022-06-01

**Authors:** Katarzyna Iłowiecka, Monika Maślej, Magdalena Czajka, Adrian Pawłowski, Piotr Więckowski, Tomasz Styk, Michał Gołkiewicz, Adam Kuzdraliński, Wojciech Koch

**Affiliations:** ^1^Department of Food and Nutrition, Medical University of Lublin, Lublin, Poland; ^2^Sundose Sp. z o.o., Lublin, Poland

**Keywords:** dietary supplements, dietary supplement users, lifestyle, dietary habits, health behaviors, characteristics, consumer surveys

## Abstract

Dietary supplements (DS) are used by about 30–50% of adults in developed countries. However, only a few studies have compared the characteristics of DS users in different nations. This study aimed to identify and compare selected health-related behaviors of DS users from three European countries. A total of 3,588 adults (32.08 ± 8.04 years) from Poland (1,030 females, 287 males), Germany (994 females, 190 males), and the United Kingdom (911 females, 176 males) were included in the analysis. The study was based on a self-administered survey consisting of 70 questions regarding baseline characteristics, lifestyle, eating, and health habits. The associations of the obtained results were compared using the Kruskal–Wallis test, Pearson Chi-Square test, and Cramer's V value. The highest percentage of DS users (56.98%, *n* = 2,044) had a correct body weight, while higher body weight values were observed in 39.19% (*n* = 1,406). In terms of lifestyle, statistically significant differences (*p* < 0.05) were noted for alcohol consumption and the level of physical activity. Fruit and vegetables were most often consumed a few times a weeks (34.67%, *n* = 1,244). A similar result was observed for the consumption of whole grain (37.76%, *n* = 1,355), dairy (39.99%, *n* = 1,435), eggs (49.67%, *n* = 1,782), and meat (51.45%, *n* = 1,846). Most DS users did not have a chronic disease (66.72%, *n* = 2,394). Among the other conditions, a frequent occurrence (a few times a weeks) of gastrointestinal problems (28.29%, *n* = 1,015) and concentration disorders (29.15%, *n* = 1,046) was noted. Cramer's V values (<0.3) indicated a weak (but significant *p* < 0.05) relationship between the country of residence and most of the analyzed variables. In conclusion, DS users were characterized by a healthy lifestyle with appropriate behaviors but not healthy eating habits.

## Introduction

According to the European Food Safety Authority (EFSA), dietary supplements (DS) can be defined as foodstuffs that are consumed for improving the standard diet. DS contain one or more concentrated ingredients such as minerals, vitamins, amino acids, herbal extracts, dietary fiber, and/or other substances with a nutritional or physiological effect. These can be easily taken orally, and are most often available in the form of capsules, tablets, pills, powders, and liquid (1). Studies indicate that at present, nearly half of the adult population in developed countries, such as the United States and Denmark, use DS (2, 3). While in other high-income countries such as Australia, South Korea, and United Arab Emirates, the proportion of DS users is around 30% (4–6). A rapid increase in the use of DS has been observed in most regions of the world over the last 35 years (7). Moreover, it has been documented that the Coronavirus pandemic has contributed to developing the DS market (8).

Following the definition, DS cannot replace a balanced healthy diet, and their consumption should depend on the nutritional status and nutrition type of individuals and populations. Results from analyzing the intake of 17 basic micronutrients in adult's diets across different European countries (including Poland, the United Kingdom, and Germany), revealed an extremely low intake of vitamin D_3_. Moreover, zinc, iron in women of childbearing age, vitamin A, and folic acid was consumed at an insufficient level (9). For this reason in some countries, recommendations regarding vitamin D supplementation have been published. National Health Service England (NHS) recommends that adults require an average of 10 μg of vitamin D3 per day and should consider taking a daily supplement during the autumn and winter months (10). The Polish expert panel represents a similar position (11). Better understanding the scope of nutrient intake adequacy across Europe is still a significant challenge.

DS are used not only to correct nutritional deficiencies but also to improve cognitive performance and overall health, prevent diseases, increase the body's efficiency, or even to extend the expected lifetime (12–14). The effectiveness of dietary supplementation in the treatment of chronic diseases is still under debate. Evidence supporting the use of vitamin and mineral supplements for the treatment or prevention of non-communicable diseases, such as cardiovascular disorders or cancer, is inadequate (15, 16). However, supplementation has been shown to be beneficial in the treatment of various commonly occurring diseases such as diabetes, obesity, and mental illness (17–19). The effect of DS can be determined by several factors, such as the type, dose, and chemical composition of the preparation, usage period, and regularity.

An important issue that gives rise to doubts both among specialists and patients regarding the use of DS is their safety. Due to the lack of a universal regulatory system, these products may contain components that are not declared on the nutrition label, such as contaminants, illegal substances, or other active ingredients capable of interacting with prescription medications or food (20, 21). Moreover, DS may affect the metabolic or physiological functions of the body. Therefore, the decision to use them should be well-considered, justified, and discussed with a health care professional. The users should routinely check the chosen products to avoid potential threats associated with their intake (20, 21).

Previous surveys on the determinants of DS have compared participants using DS and non-users (22, 23) and indicated that supplement users are more likely to be women, non-smokers, older, and have higher educational levels and incomes than people who do not use DS. Studies have shown that supplement users are typically characterized by a more healthy lifestyle, with proper eating habits, a high level of physical activity (PA), greater consumption of fruit and vegetables, and limited consumption of alcoholic beverages (24–27).

The use of DS is of interest to scientists, clinicians, and patients because these easily accessible products have a huge influence on human health. Researchers have been emphasizing the need for studies on large groups of people to understand the impact of DS on health (22). Unfortunately, the comparison of DS users within diverse populations is challenging due to the varying definitions of DS, periods of DS use, or methods of data collection (28, 29). Thus far, studies analyzing the characteristics of DS users have mostly focused on only one population (e.g., from the same country) (5, 30–33). An exception is the SENECA project (34, 35), and studies conducted by Skeie (36) and Foote (37). However, due to the rapid development of the DS market in recent years, more than 20-year-old research results need to be updated. Knowledge about the characteristics of DS users from different European countries can help to develop effective public health interventions and universal protocols for the administration of supplements (38). This study aimed to identify and compare selected health-related behaviors among the population of DS users from three European countries.

## Materials and Methods

### Study Design and Participants

This survey-based research was conducted among 3,588 DS users. A self-administered questionnaire, which was available online, was used for the study. The participants were asked to fill in the questionnaire only once. Employees of Sundose sp. z o.o. participated in the data collection and encryption.

The data for the study were collected between January 2021 and November 2021. Women and men who were using DS and filled in the survey questionnaire were eligible for the study. Additional inclusion criteria were as follows: age ≥18 years, lack of mental disorders, and willingness to provide informed consent for participation and personal data. Incomplete, unreal, or conflicting responses were rejected during the data cleaning stage (*n* = 1,728). The assumption of the data collection stage was to obtain a minimum of 1,000 results from each country. These sample size was sufficient to perform a reliable statistical analysis.

### Research Instrument

The self-administrated questionnaire consisted of 70 questions, divided into four categories:

a. Baseline information: gender (female, male); age group (18–30, 31–45, 46–60, 61–75, or >75 years); weight, height, body mass index (BMI); city population (<10.000, 10.000–50.000, 51.000–200.000, 201.000–500.000, or >500.000 people); and the purpose of supplementation (improvement of health, improvement of wellbeing, maintaining the current health state, treatment support, or better outcomes in sports).
b. Lifestyle: activity (from a few times a year to 6+ times during the week) and sport level; sleep problems and duration (from <5 to >10 h); work hours (from I don't work to >12 h); and use of drugs and stimulants.
c. Eating habits: meals quantity; snacks; most popular food categories (fruit and vegetables, whole grain, dairy, eggs, fish, meat, etc.); and food products rich in specific nutrients (e.g., avocado, fermented foods, legumes). The options provided for every question were daily, a few times a week, a few times a month, a few times a year, or no.
d. Health: use of antibiotics and medicaments; presence of skin, hair, and nail problems; diagnosis of immune disorders; diagnosis of gastrointestinal problems; and diagnosis of the most common chronic diseases such as diabetes, hypertension, anemia, osteoporosis, thyroid disease, and others.

Most of the questions required a single selection, while questions regarding age, weight, height, and stimulants required a numeric value. The survey was accessible online directly before the purchase of DS. The participants were aware that their answers define the composition of the purchased product, which made it difficult to obtain reliable data.

### Other Covariates

BMI was determined based on the World Health Organization (WHO) classification for adults (39). The value was calculated using the body weight (kg) and height (m) declared by the respondents. Based on the BMI value, the participants were categorized into the following groups: underweight, <18.50 kg/m^2^; healthy weight, 18.51–24.99 kg/m^2^; overweight, 25.00–29.99 kg/m^2^; and obese, >30.00 kg/m^2^.

### Ethical Considerations

The research protocol was approved by the Ethics Committee of Medical University of Lublin, Poland (no. KE-0254/273/2021). All subjects provided informed consent for participation in the study and collecting personal data. The analyzed data did not contain any information that could reveal the identity of the participants.

The manuscript was written following the STROBE (Strengthening the Reporting of Observational Studies in Epidemiology) guidelines for cross-sectional studies (40).

### Statistical Analysis

The obtained results were analyzed using Statistica 13.3 software (StatSoft, Inc., Tulsa, OK, United States). Categorical variables were presented as numbers and percentages, or as mean ± standard deviation if numerical. The normal distribution of numerical data was verified using the Shapiro–Wilk test. The average values from the same gender within different countries were compared using the Kruskal–Wallis test. The association between the analyzed behaviors and the inhabited country was determined using the Pearson Chi-Square test of independence. The strength of association was analyzed by calculating Cramer's V value (41). In order to indicate predictors affecting a single DS purchase cost, a generalized linear regression model analysis was performed (GRM). Significance was set at *p* < 0.05. The random allocation of participants was performed using the Mersenne Twister algorithm.

## Results

A total of 2,935 women and 653 men participated in the study (Table 1). Most of the participants were from Poland (36.71%). The percentage of women was higher compared to men for each analyzed country.

**Table 1:** Characteristics of the study group.

**Overall (%)**	**All participants**	**Poland (*n* = 1,317)**		**Germany (*n* = 1,184)**		**United Kingdom (*n* = 1,087)**	
		**Female**	**Male**	**Female**	**Male**	**Female**	**Male**
	***n* = 3,588**	**1,030 (78.21)^**B**^**	**287 (21.79)^**a**^**	**994 (83.95)^**A**^**	**190 (16.05)^**b**^**	**911 (83.81)^**A**^**	**176 (16.19)^**b**^**
**Age** **±SD (years)**	32.08 ± 8.04	30.99^**B**^ ± 8.17	33.33^**b**^ ± 8.68	32.41^**A**^ ± 8.55	34.44^**ab**^ ± 8.35	31.67^**A**^ ± 6.96	34.20^**a**^ ± 6.69
18–30	1,778 (49.55)	580 (56.31)^**A**^	132 (45.99)^**a**^	482 (48.49)^**B**^	70 (36.84)^**a**^	456 (50.05)^**AB**^	58 (32.95)^**a**^
31–45	1,595 (44.45)	393 (38.16)^**B**^	129 (44.95)^**b**^	441 (44.37)^**A**^	97 (51.05)^**ab**^	425 (46.65)^**A**^	110 (62.50)^**a**^
46–60	183 (5.10)	46 (4.47)^**A**^	22 (7.67)^**a**^	61 (6.14)^**A**^	22 (11.58)^**a**^	24 (2.63)^**A**^	8 (4.55)^**a**^
61–75	29 (0.81)	10 (0.97)^**A**^	4 (1.39)^**a**^	8 (0.80)^**A**^	1 (0.53)^**a**^	6 (0.66)^**A**^	0 (0.00)^**a**^
>75	3 (0.08)	1 (0.10)^**A**^	0 (0.00)^**a**^	2 (0.20)^**A**^	0 (0.00)^**a**^	0 (0.00)^**A**^	0 (0.00)^**a**^
**Weight** **±SD (kg)**	71.75 ± 16.99	66.50^**C**^ ± 14.09	85.09^**a**^ ± 13.62	70.84^**A**^ ± 17.27	87.35^**a**^ ± 15.61	68.50^**B**^ ± 16.07	85.77^**a**^ ± 13.27
**High** **±SD (cm)**	169.32 ± 8.14	166.97^**A**^ ± 5.90	180.48^**a**^ ± 6.49	167.62^**A**^ ± 6.38	181.02^**a**^ ± 6.41	166.13^**B**^ ± 6.12	179.59^**a**^ ± 7.28
**BMI value** **±SD (kg/m**^**2**^**)**	24.91 ± 5.24	23.80^**B**^ ± 4.59	26.11^**a**^ ± 3.97	25.21^**A**^ ± 5.96	26.64^**a**^ ± 4.41	24.80^**A**^ ± 5.56	26.57^**a**^ ± 3.68
<18.5	138 (3.84)	58 (5.63)^**A**^	2 (0.70)^**a**^	37 (3.72)^**A**^	2 (1.05)^**a**^	38 (4.17)^**A**^	1 (0.57)^**a**^
18.5–24.9	20,44 (56.98)	663 (64.37)^**A**^	127 (44.25)^**a**^	573 (57.65)^**B**^	76 (40.00)^**a**^	545 (59.82)^**AB**^	60 (34.09)^**a**^
25.0–29.9	899 (25.06)	199 (19.32)^**A**^	118 (41.11)^**a**^	204 (20.52)^**A**^	75 (39.47)^**a**^	216 (23.71)^**A**^	87 (49.43)^**a**^
>30.0	507 (14.13)	110 (10.68)^**B**^	40 (13.94)^**a**^	180 (18.11)^**A**^	37 (19.47)^**a**^	112 (12.29)^**AB**^	28 (15.91)^**a**^
**City population (thous.)**							
<10	692 (19.29)	238 (23.11)^**A**^	58 (20.21)^**a**^	271 (27.26)^**A**^	51 (26.84)^**a**^	63 (6.92)^**B**^	11 (6.25)^**b**^
10–50	673 (18.76)	185 (17.96)^**AB**^	39 (13.59)^**b**^	230 (23.14)^**A**^	51 (26.84)^**a**^	144 (15.81)^**B**^	24 (13.64)^a**b**^
51–200	841 (23.44)	198 (19.22)^**B**^	61 (21.25)^**a**^	206 (20.72)^**B**^	37 (19.47)^**a**^	281 (30.85)^**A**^	58 (32.95)^**a**^
201–500	498 (13.88)	122 (11.84)^**B**^	36 (12.54)^**a**^	88 (8.85)^**B**^	16 (8.42)^**a**^	199 (21.84)^**A**^	37 (21.02)^**a**^
>500	884 (24.64)	287 (27.86)^**A**^	93 (32.40)^**a**^	199 (20.02)^**B**^	35 (18.42)^**b**^	224 (24.59)^**AB**^	46 (26.14)^**ab**^
**Supplementation purpose**							
Health improvement	1,563 (43.56)	387 (37.57)^**B**^	95 (33.10)^**b**^	463 (46.58)^**A**^	96 (50.53)^**a**^	443 (48.63)^**A**^	79 (44.89)^**ab**^
Improvement of wellbeing	1,774 (49.44)	576 (55.92)^**A**^	150 (52.26)^**a**^	461 (46.38)^**B**^	76 (40.00)^**b**^	434 (47.64)^**B**^	77 (43.75)^**ab**^
Maintaining current state	53 (1.48)	12 (1.17)^**A**^	4 (1.39)^**a**^	19 (1.91)^**A**^	7 (3.68)^**a**^	5 (0.55)^**A**^	6 (3.41)^**a**^
Treatment support	65 (1.81)	33 (3.20)^**A**^	2 (0.70)^**a**^	15 (1.51)^**A**^	0 (0.00)^**a**^	15 (1.65)^**A**^	0 (0.00)^**a**^
Better results in sport	133 (3.71)	22 (2.14)^**A**^	36 (12.54)^**a**^	36 (3.62)^**A**^	11 (5.79)^**a**^	14 (1.54)^**A**^	14 (7.95)^**a**^

*^ABC^Values with different letters in the same row are significantly different at p <0.05 in the female group (Kruskal–Wallis test). ^abc^Values with different letters in the same row are significantly different at p <0.05 in the male group (Kruskal–Wallis test).*

*BMI, body mass index*.

Most DS users were in the 18–30 and 31–45-year age group, and the average age of the participants was 32.08 ± 8.04 years. Women and men from Poland were characterized by a significantly lower age than the participants of other countries. The analysis of BMI revealed that the highest percentage of respondents (56.98%, *n* = 2,044) had a correct body weight, while 39.19% (*n* = 1,406) of participants had an excessive body weight, of which 14.13% (*n* = 507) were obese and 25.06% (*n* = 899) were overweight. The Polish women had a statistically significantly lower body weight and BMI than the German and British women. A similar trend was observed for the men group. The data presented in Table 1 proved that most of the participants were from medium-sized (with 201.000–500.000 people) or large cities (with >500.000 people). The largest agglomerations were mostly inhabited by Polish and British than German DS users. Improvement of wellbeing (49.44%, *n* = 177) and health (43.56%, *n* = 1,563) were the primary purposes of supplementation, while only 1.48% (*n* = 53) of the study group declared that they used DS for maintaining the current health state. Table 2 presents the results of the analysis of lifestyle behaviors in the study group.

**Table 2:** Lifestyle characteristics of DS users.

**Overall (%)**	**All participants**	**Poland (*n* = 1,317)**		**Germany (*n* = 1,184)**		**United Kingdom (*n* = 1,087)**	
		**Female**	**Male**	**Female**	**Male**	**Female**	**Male**
	***n* = 3,588**	**1,030 (78.21)^**B**^**	**287 (21.79)^**a**^**	**994 (83.95)^**A**^**	**190 (16.05)^**b**^**	**911 (83.81)^**A**^**	**176 (16.19)^**b**^**
**Activity amount**							
<1x/week	1,070 (29.82)	355 (34.47)^**A**^	74 (25.78)^**a**^	274 (27.57)^**B**^	43 (22.63)^**a**^	268 (29.42)^**AB**^	56 (31.82)^**a**^
1x/week	623 (17.36)	196 (19.03)^**AB**^	56 (19.51)^**a**^	209 (21.03)^**A**^	29 (15.26)^**ab**^	120 (13.17)^**B**^	13 (7.39)^**b**^
2–3x/week	1,087 (30.29)	307 (29.81)^**A**^	74 (25.78)^**a**^	313 (31.49)^**A**^	61 (32.11)^**a**^	279 (30.63)^**A**^	53 (30.11)^**a**^
4–5x/week	626 (17.45)	126 (12.23)^**B**^	62 (21.60)^**a**^	158 (15.89)^**AB**^	38 (20.00)^**a**^	199 (21.84)^**A**^	43 (24.43)^**a**^
6+/week	182 (5.07)	46 (4.47)^**A**^	21 (7.32)^**a**^	40 (4.02)^**A**^	19 (10.00)^**a**^	45 (4.94)^**A**^	11 (6.25)^**a**^
**Training sport**							
Yes	1,957 (54.54)	518 (50.29)^**A**^	178 (62.02)^**a**^	539 (54.23)^**A**^	120 (63.16)^**a**^	507 (55.65)^**A**^	95 (53.98)^**a**^
No	1631 (45.46)	512 (49.71)^**A**^	109 (37.98)^**a**^	455 (45.77)^**A**^	70 (36.84)^**a**^	404 (44.35)^**A**^	81 (46.02)^**a**^
**Sport level**							
Amateur	851 (23.72)	228 (22.14)^**A**^	87 (30.31)^**a**^	202 (20.32)^**A**^	55 (28.95)^**a**^	242 (26.56)^**A**^	37 (21.02)^**a**^
Recreational	1,023 (28.51)	275 (26.70)^**A**^	77 (26.83)^**a**^	319 (32.09)^**A**^	55 (28.95)^**a**^	249 (27.33)^**A**^	48 (27.27)^**a**^
Competitive	83 (2.31)	15 (1.56)^**A**^	14 (4.8)^**a**^	18 (1.81)^**A**^	10 (5.26)^**a**^	16 (1.76)^**A**^	10 (5.68)^**a**^
**Sleep problems**							
Daily	431 (12.01)	120 (11.65)^**A**^	32 (11.15)^**a**^	125 (12.58)^**A**^	17 (8.95)^**a**^	120 (13.17)^**A**^	17 (9.66)^**a**^
A few/week	914 (25.47)	252 (24.47)^**A**^	69 (24.04)^**a**^	240 (24.14)^**A**^	41 (21.58)^**a**^	263 (28.87)^**A**^	49 (27.84)^**a**^
A few/month	710 (19.79)	209 (20.29)^**A**^	48 (16.72)^**a**^	207 (20.82)^**A**^	38 (20.00)^**a**^	181 (19.87)^**A**^	27 (15.34)^**a**^
A few/year	56 (1.56)	14 (1.36)^**A**^	7 (2.44)^**a**^	15 (1.51)^**A**^	1 (0.53)^**a**^	18 (1.98)^**A**^	1 (0.57)^**a**^
No	1,477 (41.16)	435 (42.23)^**A**^	131 (45.64)^**a**^	407 (40.95)^**A**^	93 (48.95)^**a**^	329 (36.11)^**A**^	82 (46.59)^**a**^
**Sleep hours**							
<5 h	81 (2.26)	20 (1.94)^**A**^	7 (2.44)^**a**^	22 (2.21)^**A**^	3 (1.58)^**a**^	25 (2.74)^**A**^	4 (2.27)^**a**^
5–6 h	1,107 (30.85)	299 (29.03)^**A**^	96 (33.45)^**a**^	293 (29.48)^**A**^	60 (31.58)^**a**^	287 (31.50)^**A**^	72 (40.91)^**a**^
7–8 h	2,161 (60.23)	634 (61.55)^**A**^	167 (58.19)^**a**^	612 (61.57)^**A**^	121 (63.68)^**a**^	534 (58.62)^**A**^	93 (52.84)^**a**^
9–10 h	223 (6.22)	74 (7.18)^**A**^	14 (4.88)^**a**^	62 (6.24)^**A**^	5 (2.63)^**a**^	61 (6.70)^**A**^	7 (3.98)^**a**^
>10 h	16 (0.45)	3 (0.29)^**A**^	3 (1.05)^**a**^	5 (0.50)^**A**^	1 (0.53)^**a**^	4 (0.44)^**A**^	0 (0.00)^**a**^
**Work hours**							
I don't work	361 (10.06)	142 (13.79)^**A**^	10 (3.48)^**a**^	115 (11.57)^**AB**^	1 (0.53)^**a**^	90 (9.88)^**B**^	3 (1.70)^**a**^
<8	585 (16.30)	167 (16.21)^**A**^	31 (10.80)^**a**^	202 (20.32)^**A**^	16 (8.42)^**a**^	154 (16.90)^**A**^	15 (8.52)^**a**^
8	1,271 (35.42)	429 (41.65)^**A**^	88 (30.66)^**a**^	341 (34.31)^**B**^	65 (34.21)^**a**^	300 (32.93)^**B**^	48 (27.27)^**a**^
9–10	877 (24.44)	188 (18.25)^**B**^	99 (34.49)^**a**^	238 (23.94)^**AB**^	70 (36.84)^**a**^	225 (24.70)^**A**^	57 (32.39)^**a**^
11–12	354 (9.87)	79 (7.67)^**A**^	37 (12.89)^**a**^	75 (7.55)^**A**^	27 (14.21)^**a**^	98 (10.76)^**A**^	38 (21.59)^**a**^
12+	140 (3.90)	25 (2.43)^**A**^	22 (7.67)^**a**^	23 (2.31)^**A**^	11 (5.79)^**a**^	44 (4.83)^**A**^	15 (8.52)^**a**^
**Daily cigarettes intake**							
No	2,848 (79.37)	848 (82.33)^**A**^	203 (70.73)^**a**^	798 (80.28)^**A**^	130 (68.42)^**a**^	740 (81.23)^**A**^	129 (73.30)^**a**^
<5	280 (7.80)	81 (7.86)^**A**^	32 (11.15)^**a**^	65 (6.54)^**A**^	13 (6.84)^**a**^	78 (8.56)^**A**^	11 (6.25)^**a**^
5–10	250 (6.97)	65 (6.31)^**A**^	18 (6.27)^**a**^	74 (7.44)^**A**^	17 (8.95)^**a**^	57 (6.26)^**A**^	19 (10.80)^**a**^
11–20	174 (4.85)	32 (3.11)^**A**^	29 (10.10)^**a**^	46 (4.63)^**A**^	16 (8.42)^**a**^	35 (3.84)^**A**^	16 (9.09)^**a**^
20+	36 (1.00)	4 (0.39)^**A**^	5 (1.74)^**a**^	11 (1.11)^**A**^	14 (7.37)^**a**^	1 (0.11)^**A**^	1 (0.57)^**a**^
**Alcohol consumption**							
Yes	2,254 (62.82)	632 (61.36)^**A**^	230 (80.14)^**a**^	556 (55.94)^B^	135 (71.05)^**a**^	575 (63.12)^**A**^	126 (71.59)^**a**^
No	1,334 (37.18)	398 (38.64)^**AB**^	57 (19.86)^**a**^	438 (44.06)^**A**^	55 (28.95)^**a**^	336 (36.88)^**B**^	50 (28.41)^**a**^
Weekly alcohol intake*							
Beer (bottle 500 ml)	1.58 ± 2.80	0.65^**A**^ ± 1.53	2.21^**a**^ ± 3.03	0.43^**B**^ ± 1.29	2.90^**a**^ ± 4.77	0.69^**A**^ ± 1.62	2.80^**a**^ ± 4.80
Wine (glass 175 ml)	1.06 ± 2.38	1.20^**A**^ ± 2.21	1.12^**a**^ ± 2.64	1.13^**A**^ ± 2.26	1.15^**ab**^ ± 3.12	1.06^**A**^ ± 2.18	0.57^**b**^ ± 1.40
Vodka (glass 50 ml)	1.20 ± 2.93	0.58^**A**^ ± 2.09	2.60^**a**^ ± 4.84	0.31^**B**^ ± 1.31	1.52^**b**^ ± 3.93	0.53^**A**^ ± 1.74	1.30^**b**^ ± 2.55
**Daily coffeecups intake**							
No	608 (16.95)	169 (16.41)^A^	51 (17.77)^**a**^	182 (18.31)^A^	27 (14.21)^**a**^	153 (16.79)^**A**^	26 (14.77)^**a**^
Occasionally	281 (7.83)	82 (7.96)^A^	25 (8.71)^**a**^	73 (7.34)^A^	13 (6.84)^**a**^	71 (7.79)^A^	17 (9.66)^**a**^
1	814 (22.69)	253 (24.56)^A^	52 (18.12)^**a**^	217 (21.83)^A^	29 (15.26)^**a**^	216 (23.71)^A^	47 (26.70)^**a**^
2–3	1,603 (44.68)	466 (45.24)^A^	121 (42.16)^**a**^	435 (43.76)^A^	86 (45.26)^**a**^	423 (46.43)^A^	72 (40.91)^**a**^
4–5	236 (6.58)	56 (5.44)^AB^	32 (11.15)^**a**^	70 (7.04)^A^	30 (15.79)^**a**^	34 (3.73)^B^	14 (7.95)^**a**^
5+	46 (1.28)	4 (0.39)^A^	6 (2.09)^**a**^	17 (1.71)^A^	5 (2.63)^**a**^	14 (1.54)^A^	0 (0.00)^**a**^

*^ABC^Values with different letters in the same row are significantly different at p <0.05 in the female group (Kruskal–Wallis test). ^abc^Values with different letters in the same row are significantly different at p <0.05 in the male group (Kruskal–Wallis test).*

*Amount of alcohol per portion.

Analysis of selected lifestyle elements was also carried out. No significant differences in gender and country of residence were observed among participants in terms of playing a sport. Among DS users who were involved in sports training (54.54%, *n* = 1,957), the largest percentage declared recreational level (28.51%, *n* = 1,023). Additional PA was rarely performed (<1 time a week, 29.32%), or the frequency was average (2–3 times a week, 30.29%). The responses showed that the number of sleep hours and sleep problems did not differ among the participants. Most participants (60.23%, *n* = 2,161) indicated that they slept 7–8 h a day and did not declare sleep problems (41.16%, *n* = 1,477). The analysis of differences with respect to country revealed no statistically significant differences in cigarette smoking and daily coffee intake among participants. On the other hand, significant differences were observed for alcohol consumption. In the group of women, the largest percentage of abstainers (44.06%, *n* = 438) were from Germany, who also consumed a significantly less amount of beer and vodka than their counterparts. Similarly, British male participants consumed significantly less wine and vodka than Poles and Germans. Compared to British and German participants, Polish men and women consumed the most considerable amounts of high-percent alcohol. Table 3 shows eating habits of the study group.

**Table 3:** Eating habits of the study group.

**Overall (%)**	**All participants**	**Poland (*****n*** **=** **1,317)**		**Germany (*****n*** **=** **1,184)**		**United Kingdom (*****n*** **=** **1,087)**	
		**Female**	**Male**	**Female**	**Male**	**Female**	**Male**
	***n* = 3,588**	**1,030 (78.21)^**B**^**	**287 (21.79)^**a**^**	**994 (83.95)^**A**^**	**190 (16.05)^**b**^**	**911 (83.81)^**A**^**	**176 (16.19)^**b**^**
**Meal frequency**							
1–2	530 (14.77)	74 (7.18)^**B**^	35 (12.20)^**b**^	253 (25.45)^**A**^	49 (25.79)^**a**^	95 (10.43)^**B**^	24 (13.64)^**ab**^
3	1,570 (43.76)	374 (36.31)^**B**^	123 (42.86)^**a**^	473 (47.59)^**A**^	90 (47.37)^**a**^	434 (47.64)^**A**^	76 (43.18)^**a**^
4	1,030 (28.71)	383 (37.18)^**A**^	81 (28.22)^**ab**^	201 (20.22)^**B**^	34 (17.89)^**b**^	271 (29.75)^**C**^	60 (34.09)^**a**^
5	438 (12.21)	194 (18.83)^**A**^	43 (14.98)^**a**^	63 (6.34)^**B**^	14 (7.37)^**a**^	108 (11.86)^**B**^	16 (9.09)^**a**^
≥6	20 (0.51)	5 (0.49)^**A**^	5 (1.74)^**a**^	4 (0.40)^**A**^	3 (1.58)^**a**^	3 (0.33)^**A**^	0 (0.00)^**a**^
**Snacks between meals**							
Daily	1,277 (35.59)	329 (31.94)^**B**^	68 (23.96)^**b**^	407 (40.95)^**A**^	50 (26.32)^**b**^	346 (37.98)^**A**^	77 (43.75)^**a**^
A few/weeks	1,115 (31.08)	343 (33.03)^**A**^	96 (33.45)^**ab**^	294 (29.58)^**A**^	70 (36.84)^**a**^	273 (29.97)^**A**^	39 (22.16)^**b**^
A few/months	345 (9.62)	117 (11.36)^**A**^	32 (11.15)^**a**^	83 (8.35)^**A**^	14 (7.37)^**a**^	86 (9.44)^**A**^	13 (7.39)^**a**^
A few/years	21 (0.59)	4 (0.39)^**A**^	0 (0.00)^**a**^	11 (1.11)^**A**^	0 (0.53)^**a**^	4 (0.44)^**A**^	0 (0.57)^**a**^
No	830 (23.13)	237 (23.01)^**A**^	91 (31.71)^**a**^	199 (20.02)^**A**^	55 (28.95)^**a**^	202 (22.17)^**A**^	46 (26.14)^**a**^
**Fruits and vegetables**							
I don't eat	36 (1.00)	4 (0.39)^**A**^	3 (1.05)^**a**^	11 (1.11)^**A**^	7 (3.68)^**a**^	8 (0.88)^**A**^	3 (1.70)^**a**^
A few/months	285 (7.94)	87 (8.45)^**A**^	42 (14.63)^**a**^	69 (6.94)^**A**^	32 (16.84)^**a**^	46 (5.05)^**A**^	9 (5.11)^**a**^
A few/weeks	1,244 (34.67)	380 (36.89)^**A**^	112 (39.02)^**a**^	340 (34.21)^**AB**^	76 (40.00)^**a**^	277 (30.41)^**B**^	59 (33.52)^**a**^
1x/days	1,093 (30.46)	281 (27.28)^**B**^	74 (25.78)^**a**^	376 (37.83)^**A**^	47 (24.74)^**a**^	261 (28.65)^**B**^	54 (30.68)^**a**^
A few/days	930 (25.92)	278 (26.99)^**B**^	56 (19.51)^**ab**^	198 (19.92)^**C**^	28 (14.74)^**b**^	319 (35.02)^**A**^	52 (28.98)^**a**^
**Avocado**							
Daily	67 (1.87)	5 (0.49)^**A**^	2 (0.70)^**a**^	17 (1.71)^**A**^	3 (1.58)^**a**^	38 (4.17)^**A**^	2 (1.14)^**a**^
A few/weeks	470 (13.10)	85 (8.25)^**B**^	16 (5.57)^**a**^	137 (13.78)^**B**^	11 (5.79)^**a**^	198 (21.73)^**A**^	23 (13.07)^**a**^
A few/months	1,240 (34.56)	326 (31.65)^**B**^	74 (25.78)^**a**^	366 (36.82)^**AB**^	54 (28.42)^**a**^	370 (40.61)^**A**^	50 (28.41)^**a**^
A few/years	782 (21.79)	282 (27.38)^**A**^	87 (30.31)^**a**^	215 (21.63)^**A**^	36 (18.95)^**b**^	128 (14.05)^**B**^	34 (19.32)^**b**^
No	1,029 (28.60)	332 (32.23)^**A**^	108 (37.63)^**a**^	259 (26.06)^**B**^	86 (45.26)^**a**^	177 (19.43)^**C**^	67 (38.07)^**a**^
**Legumes**							
Daily	80 (2.23)	28 (2.72)^**A**^	4 (1.39)^**a**^	12 (1.21)^**A**^	5 (2.63)^**a**^	29 (3.18)^**A**^	2 (1.14)^**a**^
A few/weeks	527 (14.69)	127 (12.33)^**B**^	35 (12.20)^**a**^	137 (13.78)^**AB**^	24 (12.63)^**a**^	174 (19.10)^**A**^	30 (17.05)^**a**^
A few/months	1,314 (36.62)	351 (34.08)^**B**^	108 (37.63)^**a**^	402 (40.44)^**A**^	70 (36.84)^**a**^	322 (35.35)^**AB**^	61 (34.66)^**a**^
A few/years	980 (27.31)	324 (31.46)^**A**^	80 (27.87)^**a**^	262 (26.36)^**AB**^	45 (23.68)^**a**^	216 (23.71)^**B**^	53 (30.11)^**a**^
No	687 (19.15)	200 (19.42)^**A**^	60 (20.91)^**a**^	181 (18.21)^**A**^	46 (24.21)^**a**^	170 (18.66)^**A**^	30 (17.05)^**a**^
**Whole grain**							
Daily	889 (24.78)	266 (25.83)^**A**^	63 (21.95)^**a**^	249 (25.05)^**A**^	54 (28.42)^**a**^	215 (23.60)^**A**^	42 (23.86)^**a**^
A few/weeks	1,355 (37.76)	378 (36.70)^**A**^	97 (33.80)^**a**^	390 (39.24)^**A**^	65 (34.21)^**a**^	360 (39.52)^**A**^	65 (36.93)^**a**^
A few/months	907 (25.28)	276 (26.80)^**A**^	78 (27.18)^**a**^	240 (24.14)^**A**^	42 (22.11)^**a**^	228 (25.03)^**A**^	43 (24.43)^**a**^
A few/years	273 (7.61)	79 (7.67)^**A**^	28 (9.76)^**a**^	70 (7.04)^**A**^	13 (6.84)^**a**^	67 (7.35)^**A**^	16 (9.09)^**a**^
No	164 (4.57)	31 (3.01)^**A**^	21 (7.32)^**a**^	45 (4.53)^**A**^	16 (8.42)^**a**^	41 (4.50)^**A**^	10 (5.68)^**a**^
**Dairy**							
Daily	1,400 (39.02)	381 (36.99)^**B**^	93 (32.40)^**a**^	449 (45.17)^**A**^	63 (33.16)^**a**^	339 (37.21)^**B**^	75 (42.61)^**a**^
A few/weeks	1,435 (39.99)	434 (42.14)^**A**^	129 (44.95)^**a**^	372 (37.42)^**A**^	89 (46.84)^**a**^	341 (37.43)^**A**^	70 (39.77)^**a**^
A few/months	504 (14.05)	150 (14.56)^**A**^	50 (17.42)^**a**^	107 (10.76)^**A**^	26 (13.68)^**a**^	148 (16.25)^**A**^	23 (13.07)^**a**^
A few/years	51 (1.42)	11 (1.07)^**A**^	3 (1.05)^**a**^	15 (1.51)^**A**^	6 (3.16)^**a**^	15 (1.65)^**A**^	1 (0.57)^**a**^
No	198 (5.52)	54 (5.24)^**A**^	12 (4.18)^**a**^	51 (5.13)^**A**^	6 (3.16)^**a**^	68 (7.46)^**A**^	7 (3.98)^**a**^
**Eggs (two pieces)**							
Daily	269 (7.50)	69 (6.70)^**A**^	22 (7.67)^**a**^	51 (5.13)^**A**^	8 (4.21)^**a**^	99 (10.87)^**A**^	20 (11.36)^**a**^
A few/weeks	1,782 (49.67)	530 (51.46)^**A**^	153 (53.31)^**a**^	443 (44.57)^**A**^	89 (46.84)^**a**^	467 (51.26)^**A**^	100 (56.82)^**a**^
A few/months	1,230 (34.28)	360 (34.95)^**A**^	102 (35.54)^**a**^	393 (39.54)^**A**^	71 (37.37)^**a**^	258 (28.32)^**B**^	46 (26.14)^**a**^
A few/years	140 (3.90)	32 (3.11)^**A**^	6 (2.09)^**a**^	62 (6.24)^**A**^	13 (6.48)^**a**^	23 (2.52)^**A**^	4 (2.27)^**a**^
No	167 (4.65)	39 (3.79)^**A**^	4 (1.39)^**a**^	45 (4.53)^**A**^	9 (4.74)^**a**^	64 (7.03)^**A**^	6 (3.41)^**a**^
**Fish**							
Daily	17 (0.47)	3 (0.29)^**A**^	1 (0.35)^**a**^	6 (0.60)^**A**^	1 (0.53)^**a**^	6 (0.66)^**A**^	0 (0.00)^**a**^
A few/weeks	503 (14.02)	93 (9.03)^**B**^	25 (8.71)^**a**^	154 (15.49)^**A**^	24 (12.63)^**a**^	180 (19.76)^**A**^	27 (15.34)^**a**^
A few/months	2,025 (56.44)	607 (58.93)^**A**^	171 (61.67)^**a**^	534 (53.72)^**A**^	110 (57.89)^**a**^	495 (54.34)^**A**^	102 (57.95)^**a**^
A few/years	648 (18.06)	238 (23.11)^**A**^	56 (19.51)^**a**^	177 (17.81)^**AB**^	30 (15.79)^**a**^	114 (12.51)^**B**^	33 (18.75)^**a**^
No	395 (11.01)	89 (8.64)^**A**^	28 (9.76)^**a**^	123 (12.37)^**A**^	25 (13.16)^**a**^	116 (12.73)^**A**^	14 (7.95)^**a**^
**Meat**							
Daily	790 (22.02)	202 (19.61)^**A**^	125 (43.55)^**ab**^	118 (11.87)^**B**^	63 (33.16)^**b**^	196 (21.51)^**A**^	86 (48.86)^**a**^
A few/weeks	1,846 (51.45)	558 (54.17)^**A**^	119 (41.46)^**a**^	532 (53.52)^**A**^	95 (50.00)^**a**^	473 (51.92)^**A**^	69 (39.20)^**a**^
A few/months	510 (14.21)	142 (13.79)^**B**^	25 (8.71)^**a**^	206 (20.72)^**A**^	14 (7.37)^**a**^	111 (12.18)^**B**^	12 (6.82)^**a**^
A few/years	80 (2.23)	19 (1.84)^**A**^	4 (1.39)^**a**^	32 (3.22)^**A**^	5 (2.63)^**a**^	19 (2.09)^**A**^	1 (0.57)^**a**^
No	362 (10.09)	109 (10.58)^**A**^	14 (4.88)^**a**^	106 (10.66)^**A**^	13 (6.84)^**a**^	112 (12.29)^**A**^	8 (4.55)^**a**^
**Nuts**							
Daily	174 (4.85)	34 (3.30)^**A**^	15 (5.23)^**a**^	59 (5.94)^**A**^	9 (4.74)^**a**^	51 (5.60)^**A**^	6 (3.41)^**a**^
A few/weeks	615 (17.14)	151 (14.66)^**A**^	45 (15.68)^**a**^	166 (16.70)^**A**^	25 (13.16)^**a**^	188 (20.64)^**A**^	40 (22.73)^**a**^
A few/months	1,383 (38.55)	411 (39.90)^**A**^	102 (35.54)^**a**^	389 (39.13)^**A**^	70 (36.84)^**a**^	347 (38.09)^**A**^	64 (36.36)^**a**^
A few/years	894 (24.92)	275 (26.70)^**A**^	76 (26.48)^**a**^	251 (25.25)^**A**^	45 (23.68)^**a**^	207 (22.72)^**A**^	40 (22.73)^**a**^
No	522 (14.55)	159 (15.44)^**A**^	49 (17.09)^**a**^	129 (12.98)^**A**^	41 (21.58)^**a**^	118 (12.95)^**A**^	26 (14.77)^**a**^
**Fermented food**							
Daily	55 (1.53)	12 (1.17)^**A**^	2 (0.70)^**a**^	24 (2.41)^**A**^	5 (2.63)^**a**^	11 (1.21)^**A**^	1 (0.57)^**a**^
A few/weeks	375 (10.45)	131 (12.72)^**A**^	35 (12.20)^**a**^	61 (6.14)^**B**^	15 (7.89)^**a**^	119 (13.06)^**A**^	14 (7.95)^**a**^
A few/months	1,547 (43.16)	532 (51.65)^**A**^	148 (51.57)^**a**^	330 (33.20)^**B**^	65 (34.21)^**b**^	388 (42.59)^**C**^	84 (47.73)^**ab**^
A few/years	927 (25.84)	257 (24.95)^**A**^	71 (24.74)^**a**^	305 (30.68)^**AB**^	54 (28.42)^**a**^	197 (21.62)^**B**^	43 (24.43)^**a**^
No	684 (19.06)	98 (9.51)^**B**^	31 (10.80)^**b**^	274 (27.57)^**A**^	51 (26.84)^**a**^	196 (21.51)^**A**^	34 (19.32)^**ab**^

*^ABC^Values with different letters in the same row are significantly different at p <0.05 in the female group (Kruskal–Wallis test). ^abc^Values with different letters in the same row are significantly different at p <0.05 in the male group (Kruskal–Wallis test)*.

Among dietary factors, significant differences were observed in the number of meals consumed during the day. The DS users from Poland consumed statistically significantly more number of meals than others. Furthermore, participants from Germany and the United Kingdom more often consumed snacks every day. Fruit and vegetables were most often consumed a few times a week (34.67%, *n* = 1,244). Similarly, whole grain (37.76%, *n* = 1,355), dairy (39.99%, *n* = 1,435), eggs (49.67%, n=1782), and meat (51.45%, *n* = 1,846). Avocado (34.56%, *n* = 1,240), legumes (36.62%, *n* = 1,314), fish (56.44%, *n* = 2,025), nuts (38.55%, *n* = 1,383), and fermented food (43.16%, *n* = 1,547) were consumed only a few times a month. The Kruskal–Wallis test revealed single statistically significant differences within the study group for the consumption of whole grain, dairy, eggs, and nuts. Table 4 presents the results of the analysis of health behaviors in the study group.

**Table 4:** Health characteristics of the study group.

**Overall (%)**	**All participants**	**Poland (*****n*** **=** **1,317)**		**Germany (*****n*** **=** **1,184)**		**United Kingdom (*****n*** **=** **1,087)**	
		**Female**	**Male**	**Female**	**Male**	**Female**	**Male**
	***n* = 3,588**	**1,030 (78.21)^**B**^**	**287 (21.79)^**a**^**	**994 (83.95)^**A**^**	**190 (16.05)^**b**^**	**911 (83.81)^**A**^**	**176 (16.19)^**b**^**
**Diagnosed disease**							
No	2,394 (66.72)	637 (61.84)^**B**^	201 (70.03)^**a**^	653 (65.69)^**AB**^	135 (71.05)^**a**^	633 (69.48)^**A**^	135 (76.70)^**a**^
Anemia	101 (2.81)	26 (2.52)^**A**^	1 (0.35)^**a**^	30 (3.02)^**A**^	2 (1.05)^**a**^	42 (4.61)^**A**^	0 (0.00)^**a**^
Atherosclerosis	4 (0.11)	3 (0.29)^**A**^	0 (0.00)^**a**^	1 (0.10)^**A**^	0 (0.00)^**a**^	0 (0.00)^**A**^	0 (0.00)^**a**^
Diabetes	23 (0.64)	5 (0.49)^**A**^	2 (0.70)^**a**^	9 (0.91)^**A**^	2 (1.05)^**a**^	3 (0.33)^**A**^	2 (1.14)^**a**^
Hypertension	59 (1.64)	10 (0.97)^**A**^	12 (4.18)^**a**^	13 (1.31)^**A**^	13 (6.84)^**a**^	6 (0.66)^**A**^	5 (2.84)^**a**^
Hyperthyroidism	10 (0.28)	3 (0.29)^**A**^	0 (0.00)^**a**^	3 (0.30)^**A**^	1 (0.53)^**a**^	3 (0.33)^**A**^	0 (0.00)^**a**^
Hypothyroidism	301 (8.39)	118 (11.46)^**A**^	5 (1.74)^**a**^	103 (10.36)^**AB**^	8 (4.21)^**a**^	65 (7.14)^**B**^	2 (1.14)^**a**^
Liver disease	15 (0.42)	1 (0.10)^**A**^	4 (1.39)^**a**^	0 (0.00)^**A**^	4 (2.11)^**a**^	4 (0.44)^**A**^	2 (1.14)^**a**^
Osteoporosis	7 (0.20)	1 (0.10)^**A**^	2 (0.70)^**a**^	2 (0.20)^**A**^	0 (0.00)^**a**^	2 (0.22)^**A**^	0 (0.00)^**a**^
Rheumatism	30 (0.84)	11 (1.07)^**A**^	1 (0.35)^**a**^	10 (1.01)^**A**^	1 (0.53)^**a**^	6 (0.66)^**A**^	1 (0.57)^**a**^
Other	644 (17.95)	215 (20.87)^**A**^	59 (20.56)^**a**^	170 (17.10)^**A**^	24 (12.63)^**a**^	147 (16.14)^**A**^	29 (16.48)^**a**^
**Bowel disease**							
No	3,227 (89.94)	903 (87.67)^**A**^	262 (91.29)^**a**^	905 (91.05)^**A**^	177 (93.16)^**a**^	812 (89.13)^**A**^	168 (95.45)^**a**^
Coeliac disease	24 (0.67)	7 (0.68)^**A**^	2 (0.70)^**a**^	10 (1.01)^**A**^	0 (0.00)^**a**^	5 (0.55)^**A**^	0 (0.00)^**a**^
Crohn's disease	15 (0.42)	3 (0.29)^**A**^	0 (0.00)^**a**^	8 (0.80)^**A**^	0 (0.00)^**a**^	4 (0.44)^**A**^	0 (0.00)^**a**^
Intestinal ulcers	21 (0.59)	4 (0.39)^**A**^	2 (0.70)^**a**^	4 (0.40)^**A**^	2 (1.05)^**a**^	5 (0.55)^**A**^	4 (2.27)^**a**^
SIBO/IBS	128 (3.57)	46 (4.47)^**A**^	9 (3.14)^**a**^	10 (1.01)^**A**^	2 (1.05)^**a**^	58 (6.73)^**A**^	3 (1.70)^**a**^
Other	173 (4.82)	67 (6.50)^**A**^	12 (4.18)^**a**^	57 (5.73)^**A**^	9 (4.74)^**a**^	27 (2.96)^**A**^	1 (0.57)^**a**^
**Gastrointestinal problems (flatulence; bloating; rumbling in intestines)**							
daily	510 (14.21)	153 (14.85)^**A**^	43 (14.98)^**a**^	145 (14.59)^**A**^	22 (11.58)^**a**^	121 (13.28)^**A**^	26 (14.77)^**a**^
a few/weeks	1,015 (28.29)	301 (29.22)^**A**^	73 (25.44)^**a**^	282 (28.37)^**A**^	55 (28.95)^**a**^	261 (28.65)^**A**^	43 (24.43)^**a**^
a few/months	1,004 (27.98)	322 (31.26)^**A**^	91 (31.71)^**a**^	260 (26.16)^**A**^	38 (20.00)^**b**^	258 (28.32)^**A**^	35 (19.89)^**b**^
a few/years	195 (5.43)	58 (5.63)^**A**^	19 (6.62)^**a**^	43 (4.33)^**A**^	10 (5.26)^**a**^	57 (6.26)^**A**^	8 (4.55)^**a**^
No	864 (24.08)	196 (19.03)^**B**^	61 (21.25)^**b**^	264 (26.56)^**A**^	65 (34.21)^**a**^	214 (23.49)^**AB**^	64 (36.36)^**a**^
**Vomit/diarrhea**							
Daily	31 (0.86)	10 (0.97)^**A**^	2 (0.70)^**a**^	9 (0.91)^**A**^	2 (1.05)^**a**^	7 (0.77)^**A**^	1 (0.57)^**a**^
a few/weeks	163 (4.54)	42 (4.08)^**A**^	13 (4.53)^**a**^	54 (5.43)^**A**^	10 (5.26)^**a**^	37 (4.06)^**A**^	7 (3.98)^**a**^
a few/months	567 (15.80)	173 (16.80)^**A**^	42 (14.63)^**a**^	161 (16.20)^**A**^	32 (16.84)^**a**^	141 (15.84)^**A**^	18 (10.23)^**a**^
a few/years	498 (13.88)	155 (15.05)^**AB**^	42 (14.63)^**a**^	101 (10.16)^**B**^	13 (6.84)^**a**^	168 (18.44)^**A**^	19 (10.80)^**a**^
No	2,329 (64.91)	650 (63.11)^**A**^	188 (65.51)^**a**^	669 (67.30)^**A**^	133 (70.00)^**a**^	558 (61.25)^**A**^	131 (74.43)^**a**^
**Immune disorders (throat infection;sinusitis; flu) in the last years**							
No	1,404 (39.13)	306 (29.71)^**C**^	94 (32.75)^**b**^	496 (49.90)^**A**^	105 (55.26)^**a**^	331 (36.33)^**B**^	72 (40.91)^**ab**^
1x/season	1,162 (32.39)	367 (35.63)^**A**^	91 (31.71)^**a**^	248 (24.95)^**B**^	55 (28.95)^**a**^	337 (36.99)^**A**^	64 (36.36)^**a**^
1x/quarter	758 (21.13)	259 (25.15)^**A**^	80 (27.87)^**a**^	186 (18.71)^**B**^	23 (12.11)^**b**^	179 (19.65)^**AB**^	31 (17.61)^**ab**^
1x/months	200 (5.57)	78 (7.57)^**A**^	14 (4.88)^**a**^	47 (4.73)^**A**^	7 (3.68)^**a**^	47 (5.16)^**A**^	7 (3.98)^**a**^
> 2x/months	64 (1.78)	20 (1.94)^**A**^	8 (2.79)^**a**^	17 (1.71)^**A**^	0 (0.00)^**a**^	17 (1.87)^**A**^	2 (1.14)^**a**^
**Headaches**							
Daily	103 (2.87)	30 (2.91)^**A**^	4 (1.39)^**a**^	31 (3.12)^**A**^	4 (2.11)^**a**^	27 (2.96)^**A**^	7 (3.98)^**a**^
a few/weeks	640 (17.84)	189 (18.35)^**A**^	30 (10.45)^**a**^	214 (21.53)^**A**^	20 (10.53)^**a**^	161 (17.67)^**A**^	26 (14.77)^**a**^
a few/months	1,457 (40.61)	481 (46.70)^**A**^	88 (30.33)^**a**^	394 (39.64)^**B**^	52 (27.37)^**a**^	398 (43.69)^**AB**^	44 (25.00)^**a**^
a few/years	415 (11.57)	128 (12.43)^**A**^	47 (16.38)^**a**^	97 (9.76)^**A**^	22 (11.58)^**a**^	100 (10.98)^**A**^	21 (11.93)^**a**^
No	973 (27.12)	202 (19.61)^**B**^	118 (41.11)^**a**^	258 (25.96)^**A**^	92 (48.42)^**a**^	225 (24.70)^**AB**^	78 (44.32)^**a**^
**Concentration problems**							
Daily	591 (16.47)	170 (16.50)^**A**^	54 (18.82)^**a**^	153 (15.39)^**A**^	19 (10.00)^**a**^	166 (18.22)^**A**^	29 (16.48)^**a**^
a few/weeks	1,046 (29.15)	323 (31.36)^**A**^	85 (29.62)^**a**^	285 (28.67)^**A**^	47 (24.74)^**a**^	265 (28.10)^**A**^	50 (28.41)^**a**^
a few/months	801 (22.32)	242 (23.50)^**A**^	54 (18.82)^**a**^	235 (23.64)^**A**^	39 (20.53)^**a**^	206 (22.61)^**A**^	25 (14.20)^**a**^
a few/years	121 (3.37)	26 (2.52)^**A**^	7 (2.44)^**a**^	35 (3.52)^**A**^	6 (3.16)^**a**^	41 (4.50)^**A**^	6 (3.41)^**a**^
No	1,029 (28.68)	269 (26.12)^**A**^	87 (30.31)^**a**^	286 (28.77)^**A**^	79 (41.58)^**a**^	242 (26.56)^**A**^	66 (37.50)^**a**^
**Muscle tremors**							
Daily	134 (3.73)	37 (3.59)^**A**^	17 (5.92)^**a**^	26 (2.62)^**A**^	5 (2.63)^**a**^	38 (4.17)^**A**^	11 (6.25)^**a**^
a few/weeks	457 (12.74)	140 (13.59)^**A**^	41 (14.29)^**a**^	106 (10.66)^**A**^	24 (12.63)^**a**^	123 (13.50)^**A**^	23 (13.07)^**a**^
a few/months	1,055 (29.40)	364 (35.34)^**A**^	89 (31.01)^**a**^	259 (26.06)^**B**^	40 (21.05)^**a**^	257 (28.21)^**B**^	46 (26.14)^**a**^
a few/years	464 (12.93)	154 (14.95)^**A**^	45 (15.68)^**a**^	103 (10.36)^**A**^	19 (10.00)^**a**^	125 (13.72)^**A**^	18 (10.23)^**a**^
No	1,478 (41.19)	335 (32.52)^**C**^	95 (33.10)^**b**^	500 (50.30)^**A**^	102 (53.68)^**a**^	368 (40.40)^**B**^	78 (44.32)^**ab**^
**Skin problems(multiple choice)**							
No	821 (22.88)	161 (15.63)^**A**^	105 (36.59)^**b**^	192 (19.32)^**A**^	93 (48.95)^**a**^	185 (20.31)^**A**^	85 (48.30)^**ab**^
Acne	1,321 (36.82)	425 (41.26)^**A**^	72 (25.09)^**a**^	379 (38.13)^**A**^	37 (19.47)^**a**^	379 (41.60)^**A**^	29 (16.48)^**a**^
Discoloration	1,098 (30.60)	424 (41.17)^**A**^	46 (16.03)^**a**^	257 (25.86)^**B**^	19 (10.00)^**a**^	321 (35.24)^**A**^	31 (17.61)^**a**^
Dry and rough	1,725 (48.08)	571 (55.44)^**A**^	110 (38.33)^**a**^	524 (52.72)^**A**^	66 (34.74)^**a**^	391 (42.92)^**B**^	63 (35.80)^**a**^
Flaking	428 (11.93)	124(12.04)^**A**^	45 (15.68)^**a**^	119 (11.97)^**A**^	23 (12.11)^**a**^	87 (9.55)^**A**^	30 (17.05)^**a**^
Perleche	312 (8.70)	111 (10.78)^**A**^	28 (9.76)^**a**^	105 (10.56)^**A**^	7 (3.68)^**a**^	52 (5.71)^**B**^	9 (5.11)^**a**^
Slow wound healing	504 (14.05)	163 (15.83)^**A**^	17 (5.92)^**a**^	147 (14.79)^**A**^	14 (7.37)^**a**^	146 (16.03)^**A**^	17 (9.66)^**a**^
Keratosis	474 (13.21)	140 (13.59)^**AB**^	47 (16.38)^**a**^	184 (18.51)^**A**^	14 (7.37)^**a**^	78 (8.56)^**B**^	11 (6.25)^**a**^
Other	422 (11.76)	104 (10.10)^**A**^	21 (7.32)^**a**^	130 (13.08)^**A**^	18 (9.47)^**a**^	136 (14.93)^**A**^	13 (7.39)^**a**^
**Hair problems (multiple choice)**							
No	1,082 (30.16)	211 (20.49)^**B**^	167 (58.19)^**a**^	284 (28.57)^**A**^	111 (58.42)^**a**^	210 (23.05)^**AB**^	99 (56.25)^**a**^
Dandruff	642 (17.89)	194 (18.83)^**A**^	41 (14.29)^**a**^	190 (19.11)^**A**^	23 (12.11)^**a**^	169 (18.55)^**A**^	25 (14.20)^**a**^
Dry and brittle	1,641 (45.74)	568 (55.15)^**A**^	32 (11.15)^**a**^	511 (51.41)^**A**^	26 (13.68)^**a**^	485 (53.24)^**A**^	19 (10.80)^**a**^
Loss	1,981 (55.21)	677 (65.73)^**B**^	89 (31.01)^**a**^	526 (52.92)^**A**^	58 (30.53)^**a**^	580 (63.67)^**B**^	51 (28.98)^**a**^
Matt	1,013 (28.23)	361 (35.05)^**A**^	25 (8.71)^**a**^	295 (29.68)^**A**^	16 (8.42)^**a**^	299 (32.82)^**A**^	17 (9.66)^**a**^
Other	337 (9.39)	94 (9.13)^**A**^	19 (6.62)^**a**^	103 (10.36)^**A**^	14 (7.37)^**a**^	92 (10.10)^**A**^	15 (8.52)^**a**^
**Nail problems (multiple choice)**							
No	1,700 (47.38)	393 (38.16)^**A**^	214 (74.56)^**a**^	476 (47.89)^**B**^	146 (76.84)^**a**^	349 (38.31)^**A**^	122 (69.32)^**a**^
Fragile and brittle	1,449 (40.38)	549 (53.30)^**A**^	45 (15.68)^**a**^	371 (37.32)^**B**^	25 (13.16)^**a**^	433 (47.53)^**A**^	26 (14.77)^**a**^
Grow slowly	520 (14.49)	155 (15.05)^**A**^	6 (2.09)^**a**^	168 (16.90)^**A**^	11 (5.79)^**a**^	172 (18.88)^**A**^	8 (4.55)^**a**^
Inflammations	122 (3.40)	20 (1.94)^**A**^	8 (2.79)^**a**^	49 (4.93)^**A**^	7 (3.68)^**a**^	32 (3.51)^**A**^	6 (3.41)^**a**^
Splitting	1,066 (29.71)	343 (33.30)^**A**^	27 (9.41)^**a**^	314 (31.59)^**A**^	17 (8.95)^**a**^	343 (37.65)^**A**^	22 (12.50)^**a**^
White spots	562 (15.66)	170 (16.50)^**A**^	27 (9.41)^**a**^	154 (15.49)^**A**^	13 (6.84)^**a**^	170 (18.66)^**A**^	28 (15.91)^**a**^
Other	148 (4.12)	26 (2.52)^**A**^	12 (4.18)^**a**^	51 (5.13)^**A**^	7 (3.68)^**a**^	40 (4.39)^**A**^	12 (6.82)^**a**^
**Medicaments (multiple choice)**							
No	2,443 (68.09)	636 (61.75)^**B**^	227 (79.09)^**a**^	650 (65.39)^**AB**^	147 (77.37)^**a**^	643 (70.58)^**A**^	140 (79.54)^**a**^
Anticancer	5 (0.14)	0 (0.00)^**A**^	0 (0.00)^**a**^	4 (0.40)^**A**^	0 (0.00)^**a**^	1 (0.11)^**A**^	0 (0.00)^**a**^
Antiepileptic	10 (0.28)	5 (0.49)^**A**^	0 (0.00)^**a**^	5 (0.50)^**A**^	0 (0.00)^**a**^	0 (0.00)^**A**^	0 (0.00)^**a**^
Contraceptives	367 (10.23)	135 (13.11)^**A**^	0 (0.00)^**a**^	128 (12.88)^**A**^	0 (0.00)^**a**^	104 (11.42)^**A**^	0 (0.00)^**a**^
Diuretics	16 (0.45)	7 (0.68)^**A**^	4 (1.39)^**a**^	5 (0.50)^**A**^	0 (0.00)^**a**^	0 (0.00)^**A**^	0 (0.00)^**a**^
For stomach acidity	62 (1.73)	17 (1.65)^**A**^	7 (2.44)^**a**^	18 (1.81)^**A**^	5 (2.63)^**a**^	9 (0.99)^**A**^	6 (3.41)^**a**^
Metformin	42 (1.17)	23 (2.23)^**A**^	1 (0.35)^**a**^	9 (0.91)^**A**^	2 (1.05)^**a**^	5 (0.55)^**A**^	2 (1.14)^**a**^
Salicylates	8 (0.22)	3 (0.29)^**A**^	1 (0.35)^**a**^	3 (0.30)^**A**^	0 (0.00)^**a**^	1 (0.11)^**A**^	0 (0.00)^**a**^
Steroids	40 (1.11)	20 (1.94)^**A**^	4 (1.39)^**a**^	4 (0.40)^**A**^	3 (1.58)^**a**^	6 (0.66)^**A**^	3 (1.70)^**a**^
Chelation therapy	1 (0.03)	0 (0.00)^**A**^	0 (0.00)^**a**^	1 (0.10)^**A**^	0 (0.00)^**a**^	0 (0.00)^**A**^	0 (0.00)^**a**^
Other	773 (21.54)	254 (24.66)^**A**^	51 (17.77)^**a**^	231 (23.24)^**A**^	35 (18.42)^**a**^	175 (19.21)^**A**^	27 (15.34)^**a**^
**Antibiotics in the last 4 years**							
No	1,306 (36.40)	285 (27.67)^**B**^	92 (32.06)^**b**^	367 (36.92)^**A**^	93 (48.95)^**a**^	373 (40.94)^**A**^	96 (54.55)^**a**^
1–2x	1,434 (39.97)	441 (42.82)^**A**^	117 (40.77)^**a**^	390 (39.24)^**A**^	75 (39.47)^**a**^	354 (38.86)^**A**^	57 (32.39)^**a**^
3–4x	575 (16.03)	199 (19.32)^**A**^	47 (16.38)^**a**^	158 (15.90)^**A**^	13 (6.84)^**a**^	139 (15.26)^**A**^	19 (10.80)^**a**^
5+	273 (7.61)	105 (10.19)^**A**^	31 (10.80)^**a**^	79 (7.95)^**A**^	9 (4.74)^**a**^	45 (4.49)^**A**^	4 (2.27)^**a**^

*^ABC^Values with different letters in the same row are significantly different at p <0.05 in the female group (Kruskal–Wallis test). ^abc^Values with different letters in the same row are significantly different at p <0.05 in the male group (Kruskal–Wallis test).*

*SIBO, small intestinal bacterial overgrowth; IBS, irritable bowel syndrome*.

The results indicated that most of the DS users did not have any diagnosed chronic disease, while the remaining suffered from hypothyroidism (8.39%, *n* = 309) or anemia (2.81%, *n* = 101). The amount and type of medications used by these participants corresponded with these diagnosed conditions. A considerable number of participants declared frequent occurrence (a few times a week) of gastrointestinal problems (28.29%, *n* = 1,015) and concentration disorders (29.15%, *n* = 1,046), while headaches (40.61%, *n* = 1,457), and muscle tremors (29.40%, *n* = 1,055) were reported slightly less frequently (a few times a month). Immune disorders were relatively rare (39.13% (*n* = 1,404) did not have them in the last year), and the frequency of antibiotic use was also very less. The most frequently reported skin, hair, and nail conditions were, respectively, dry/rough (48.08%, *n* = 1,725), loss (55.21%, *n* = 1,981), and fragile/brittle (40.38%, *n* = 1,449). Regarding health characteristics, the men group was more homogeneous than females.

Table 5 presents the association between the analyzed behaviors and the country of residence in the study group.

**Table 5:** Association between the analyzed behaviors and the country of residence in the study group.

**Variables**	**Pearson Chi-Square(*X*^2^)**	***p*-Value**	**Cramer's V value**
BMI	34.22	0.00001	0.07
City population	291.82	0.00000	0.20
Supplementation purpose	57.46	0.00000	0.09
Activity level	55.09	0.00000	0.09
Training sport	2.43	0.29668	0.26
Sport level	11.16	0.08325	0.04
Sleep problems	12.75	0.12073	0.04
Sleep hours	6.67	0.57218	0.03
Work hours	46.91	0.00000	0.08
Daily cigarettes intake	29.06	0.00031	0.06
Alcohol consumption	15.28	0.00048	0.07
Daily coffeeintake	23.78	0.00250	0.06
Meals amount	281.88	0.00000	0.20
Snacks between meals	37.33	0.00001	0.10
Fruits and vegetables	96.90	0.00000	0.12
Avocado	189.85	0.00000	0.16
Legumes	38.42	0.00001	0.07
Whole grain	7.99	0.43438	0.33
Dairy	30.64	0.00016	0.07
Eggs	97.01	0.00000	0.12
Fish	83.87	0.00000	0.11
Meat	70.33	0.00000	0.10
Nuts	26.32	0.00093	0.06
Fermented food	206.69	0.00000	0.17
Bowel disease	58.84	0.00000	0.09
Gastrointestinal problems	32.15	0.00009	0.07
Vomit/diarrhea	31.93	0.00010	0.07
Immune disorders	124.93	0.00000	0.13
Headaches	21.76	0.00539	0.06
Concentration problems	16.76	0.03270	0.05
Muscle tremors	90.77	0.00000	0.11
Skin problems	51.88	0.00001	0.09
Hair problem	39.53	0.00002	0.07
Nail problem	107.64	0.00000	0.12
Antibiotics	76.44	0.00000	0.10

*BMI, body mass index*.

In consonance with the Pearson Chi-Square test requirement for independence, in at least 80% of cells, the expected value should be 5 or greater (42). Accordingly, medicaments and diagnosed diseases were excluded from the analysis.

The strength of association between variables was determined through Cramer's V tests. The values of V can range from 0 to 1. A value of 1 or 0 indicates a strong or lack of a relationship, respectively, while values <0.3 indicate a weak relationship between the analyzed variables. The results obtained in this study showed an association between most of the analyzed behaviors and the country of residence (*p* < 0.05), whereas no association was found for sports training, sport level, sleep problems, sleep hours, and whole-grain consumption (*p* > 0.05).

A tendency to invest in DS may indirectly influence the determination to use supplementation. An average single cost of a purchased DS in a study group was 15.6 €. In order to indicate predictors affecting this value, a generalized linear regression model analysis was performed. The significant (*p* < 0.05) effects and their interactions are specified in Table 6. The results indicate that the cost of single DS purchase variability depends on diverse factors—related to health characteristics, eating habits, lifestyle or demographic parameters.

**Table 6:** Generalized linear regression model (GRM) for an average cost of a purchased DS.

**Variables**	**SS**	**df**	**MS**	***F*-Value**	***p*-Value**
Antibiotics	1.25	3	1.25	5.94	0.01482
Avocado	3.79	4	0.95	4.50	0.00127
City population (thous.)	3.80	4	0.63	3.00	0.00628
Concentration problems	2.52	4	0.63	2.99	0.01784
Diagnosed disease	2.88	10	2.88	13.68	0.00022
Fermented food	4.43	4	1.11	5.26	0.00032
Fish	3.12	4	0.78	3.70	0.00520
Immune disorders	2.27	4	0.57	2.69	0.02946
Medicaments	567.62	10	567.62	2,694.40	0.00000
Muscle tremors	1.38	4	1.38	6.57	0.01044
Snacks between meals	1.36	4	1.36	6.45	0.01112
Work hours	2.63	5	0.53	2.50	0.02899
**SS-test results for the full model**	**Multiple R**	**Multiple R** ^**2**^		**Adjusted R** ^**2**^	***p*** **-Value**
	0.71999	0.51838		0.51008	0.00000

*SS, sum of squares; MS, mean square; df, degrees of freedom; R, correlation coefficient; R^2^, coefficient of determination*.

The SS-test for the presented complete model in relation to SS-test for the residues indicates that the model describes medium-well the dependent variable, as it is evidenced by a value of the determination coefficient. The R^2^ of 0.518 indicates that 51.8% of the variance of the dependent variable cost is explained by this model. Therefore, the remaining 48.2% of the variation is explained by other unidentified factors.

## Discussion

A rapid increase in the use of DS in recent years prompted us to compare selected health-related behaviors in DS users. Furthermore, only a few studies have been conducted so far among DS users from different nationalities (34–37). Thus, the present study is the first to provide detailed information on personalized DS users from Poland, Germany, and the United Kingdom.

Several research teams have compared the users and non-users of DS (5, 23, 24, 43–48). In terms of gender, the results obtained in this study are consistent with most scientific reports, in which it has been shown that DS are more often used by women. Some reports (5, 24, 44, 45, 49) suggest women predominance in the range of 5–10 percentage points, while other studies (22, 23, 34, 47, 49), including ours, indicate more significant gender discrepancy. Few studies have shown more frequent consumption of DS by men (47). The average age of the participants in the present study was 32.08 ± 8.04 years, and the largest percentage of DS users were in the age group of 18–30 years (49.55%). The obtained values are lower compared to those presented in most of the earlier studies. This may be due to the fact that in our study, the purchase of DS was mainly *via* the Internet, which is more often practiced by young people (50–52). A relationship between the use of DS and the correct BMI has been demonstrated in previous studies (5, 22, 23, 43, 49). Our study showed a normal BMI in most of the participants. However, significant differences were observed in terms of gender and country of residence. The average BMI of men and women from Germany indicated overweight (BMI > 25 kg/m^2^). Similar results have been reported for DS users from Greece (30) and Belgium (31). It is worth emphasizing that in this study the DS users group included a low percentage of obese people (14.13%), compared to domestic populations, as around 24% in Poland (53) or Germany (54) and 28% in the United Kingdom are obese (55).

The results of our investigation showed that the main purposes for DS use are improvement of health and wellbeing. Previous studies have shown that the most frequently reported reasons for DS use are “solving or overcoming health problems”(22), “health” (43), “injury or illness” (56), or “medical need/deficiency”(47). These outcomes are in contrast with the reported overall health. The findings of the present study indicated that 66.72% of participants were not diagnosed with any chronic disease. Other studies had also well-documented that most DS users are characterized by appropriate health status. Burnett et al. (5) showed a lack of chronic disease in 91.36% of participants. Similarly, Radwan et al. (47) and Rontogianni et al. (30) revealed that the majority of DS users do not suffer from persistent illnesses. On the other hand, some reports indicated a lower percentage (around 20%) of healthy adults in the studied population (44, 45). Skin, hair, and nail problems were the most common in the group analyzed in the present study. A literature search suggested that this is the first study to report numerous dermatological disorders among DS users. Previous studies have shown that “beauty” or “skin, hair, nails” is one of the primary purposes of supplementation (14, 22, 25, 43). Taking all these into account, the results of the present study confirm the expectations that the global beauty supplement market will reach $7 billion by 2024 (57). Regarding medications, our results showed that contraceptives were the most frequently used drug among the participants (10.23%). As revealed by numerous studies, the main ingredient of DS that may reduce the effectiveness of oral contraceptives and increase the risk of breakthrough bleeding is St. John's wort (58–60). Therefore, women taking oral contraceptives should pay attention to the composition of herbal supplements.

According to the WHO (61), regular PA (at least five times a week) can contribute to an improvement in biomedical markers. Our results showed that only 25.92% of DS users followed regular PA. The highest percentage of British DS users were involved in PA for 4–5 times a week, with statistically significant differences observed among women. Additionally, 10.00% of German men and 4.94% of British women were active, involving in PA >6 times a week. These results are in line with the study of Kim et al. (44), who reported that 27.79% of DS users declared a “high” level of PA. Among Australian adults, 43.00% of DS users met the national recommendations (5). Similar results were indicated by Pouchieu et al. (22) (42.50%) and Guo et al. (32) (47.90%). Another study (33) showed that only 15.30% of male and 21.30% of female DS users in Japan undertook physical exercise for 3–7 times a week. On the other hand, as much as 57.6% of Belgium army men declared a high level of PA (31).

The recommended sleep duration for adults is 7 h/day or more (62). The obtained results proved that more than 60% of DS users met this recommendation. No statistically significant differences were observed between participants in terms of country and gender. Furthermore, most of the participants (61.16%) did not have any sleep problems. Our results confirmed those reported by Dickinson et al. (14), who showed that DS users significantly more often “had a good night's sleep” compared to non-users (70 vs. 63%). Several studies concerning lifestyle characteristics have analyzed behaviors such as cigarette smoking, alcohol use, or coffee consumption among DS users. In the current study, 79.37% of participants declared no smoking (*p* > 0.05 for country and gender). This value agrees with most of the previous works, in which 75.9–88.24% of DS users have been described as ex-smokers and/or non-smokers (5, 22, 31, 32, 37, 44, 47, 63). Former studies have indicated a significant, positive association between alcohol absence and DS use (24, 63, 64). Moreover, the association with different types of alcohol separately has been widely investigated. In contrast to beer consumption, a positive relationship has been found for wine consumption (65, 66). Scientific reports mention different percentages of abstainers among DS users. For example, Rautiainen et al. (23) showed 16.4% of the study group were non-drinkers, and Rontogianni et al. (30) reported 12.21%. On the other hand, Kim et al. (44) and Guo et al. (32) showed a higher percentage of non-drinkers (41.62 and 40.9%, respectively). Our results demonstrated that 37.18% were abstainers, of which the largest (*p* < 0.05) percentage were German females (44.06%). Few works regarding coffee intake among DS users have shown an inverse relationship between supplement use and coffee consumption in the women group (66). Our research indicated that the highest percentage of DS users (44.68%) consumed reasonable amounts of coffee (about 2–3 cups a day) in each studied country.

According to previous studies (25, 37), participants practicing correct dietary habits used DS more often than those who had not been following a proper diet. The most frequently analyzed eating habit was the consumption of fruit and vegetables. Some reports proved that more than 46.00% of DS users eat ≥5 servings of fruit and vegetables daily (32, 43). Reedy et al. (45) and Beitz et al. (66) indicated that DS users consumed adequate portions of fruit and vegetables per days (5.63 and 6.71, respectively). In contrast, only 7.49% of Australian DS users met daily guidelines (67) recommending five servings of vegetables and two servings of fruit (5). Our results revealed that 25.92% of respondents consumed a sufficient amount of vegetables and fruit (a few times a days). Significant differences were observed with respect to gender and country (the most favorable results were obtained for British DS users). The other plant-based foods (legumes, nuts, avocado) were most often consumed several times a months. It is recommended that legumes should be consumed 2–3 times a week (68) and unsalted nuts should be consumed four times a week (or 30 g daily) (69). However, our study showed that only 14.69% (for legumes) and 17.14% (for nuts) of the participants met these guidelines. Similarly, less than one-fourth of the study group (24.78%) consumed whole grain daily, as per the recommendations (70). Animal products (dairy, eggs, meat) were most often consumed a few times a week which is also in line with the guidelines (71–73). In contrast, the frequency of fish consumption by most of the studied DS users was insufficient (74).

Our results indicated that the number of meals consumed during the day significantly differed in the study group. Female DS users from Poland often consumed significantly more number [4 (37.18%) or 5 (18.83%)] of meals compared to Germans and British women. On the other hand, no differences were observed based on nationalities in the group of men. The largest percentage (over 40.00%) consumed three meals a day, which is less than the current Polish or German recommendations (4–5 meals) (75, 76) but in accordance with the British guidelines (at least three meals) (77).

The study has several strengths. First, the analysis of data from different countries allowed for a more comprehensive consideration of dietary supplementation issues. Second, all participants acquired DS from the same source and in a similar form. This enabled eliminating variations in the definition of supplement users, which was an issue in several previous studies (28, 29, 43, 66). A wide range of data was analyzed, contributing to a comprehensive assessment of the most common health behaviors. Furthermore, this research was conducted by an interdisciplinary team consisting of dietitians (KI., MM., TS.), biotechnologists (AK., MC., AP.), and pharmacists (WK.), by applying a multidimensional approach.

Although further studies on a larger number of participants are needed, the sample of DS users analyzed in this study is similar to those investigated in some previous studies on this topic (5, 30, 31, 48, 49). A limitation of the study is the use of a self-administered questionnaire, which might cause errors in data collection. Moreover, the study could not collect detailed information on the size of food portions, components of applied supplements, or health measures. However, similar results have been obtained in several previous works.

## Conclusions

In summary, the obtained results demonstrate that DS users had a proper health state associated with healthy lifestyle behaviors. Most participants were characterized by a correct BMI, limited smoking and alcohol consumption, a rare occurrence of diseases, and sufficient sleep. However, the consumption of fruit and vegetables, nuts, legumes, and fish was low and meal frequency was inadequate in the study group. Most of the analyzed behaviors showed an association with nationality.

## Data Availability Statement

The datasets presented in this article are not readily available because of ethics restrictions. Requests to access the datasets should be directed to KI, katarzyna.ilowiecka@umlub.pl.

## Ethics Statement

The studies involving human participants were reviewed and approved by Ethics Committee of Medical University of Lublin. The patients/participants provided their written informed consent to participate in this study.

## Author Contributions

KI: conceptualization and writing review and editing. KI and WK: methodology and writing manuscript. PW, KI, and AK: data analysis. KI, AK, WK, MC, MM, AP, TS, MG, and PW: resources. WK: supervision. All authors have made a substantial, direct, and intellectual contribution to the work and have approved to the final version of the manuscript.

## Conflict of Interest

MM, MC, AP, PW, TS, MG, and AK was employed by Sundose Sp. z o.o. The remaining authors declare that the research was conducted in the absence of any commercial or financial relationships that could be construed as a potential conflict of interest.

## Publisher's Note

All claims expressed in this article are solely those of the authors and do not necessarily represent those of their affiliated organizations, or those of the publisher, the editors and the reviewers. Any product that may be evaluated in this article, or claim that may be made by its manufacturer, is not guaranteed or endorsed by the publisher.
